# Building a Robust Tumor Profiling Program: Synergy between Next-Generation Sequencing and Targeted Single-Gene Testing

**DOI:** 10.1371/journal.pone.0152851

**Published:** 2016-04-04

**Authors:** Matthew C. Hiemenz, Stephan Kadauke, David B. Lieberman, David B. Roth, Jianhua Zhao, Christopher D. Watt, Robert D. Daber, Jennifer J. D. Morrissette

**Affiliations:** 1 Center for Personalized Diagnostics, Department of Pathology and Laboratory Medicine, Hospital of the University of Pennsylvania, Philadelphia, Pennsylvania, United States of America; 2 Medical Scientist Training Program, Perelman School of Medicine at the University of Pennsylvania, Philadelphia, Pennsylvania, United States of America; National Institute of Technology, Rourkela, INDIA

## Abstract

Next-generation sequencing (NGS) is a powerful platform for identifying cancer mutations. Routine clinical adoption of NGS requires optimized quality control metrics to ensure accurate results. To assess the robustness of our clinical NGS pipeline, we analyzed the results of 304 solid tumor and hematologic malignancy specimens tested simultaneously by NGS and one or more targeted single-gene tests (*EGFR*, *KRAS*, *BRAF*, *NPM1*, *FLT3*, and *JAK2*). For samples that passed our validated tumor percentage and DNA quality and quantity thresholds, there was perfect concordance between NGS and targeted single-gene tests with the exception of two *FLT3* internal tandem duplications that fell below the stringent pre-established reporting threshold but were readily detected by manual inspection. In addition, NGS identified clinically significant mutations not covered by single-gene tests. These findings confirm NGS as a reliable platform for routine clinical use when appropriate quality control metrics, such as tumor percentage and DNA quality cutoffs, are in place. Based on our findings, we suggest a simple workflow that should facilitate adoption of clinical oncologic NGS services at other institutions.

## Introduction

The advance of next-generation sequencing (NGS) is a cornerstone of a recent development in molecular pathology, variably referred to as “personalized,” “precision,” or “individualized” medicine. Much of the focus of clinical NGS has been on oncology, as there are clear diagnostic, prognostic, and therapeutic implications for a multitude of genomic mutations in both solid and liquid malignancies. For example, in non-small cell lung cancers, activating mutations of the *EGFR* gene predict therapeutic response to tyrosine kinase inhibitors (TKI) such as erlotinib, gefitinib, and afatinib [1–3]. The KRAS protein acts downstream of EGFR, and thus mutations of the *KRAS* gene predict resistance to TKI [2,4–6]. In metastatic melanoma, *BRAF* V600 mutations predict response to dabrafenib, vemurafenib, and trametinib [7]. Mutations of the *FLT3* and *NPM1* genes affect the prognosis of karyotypically normal acute myeloid leukemia and aid in the decision whether or not to pursue hematopoietic stem cell transplantation [8]. In addition, activating mutations of *JAK2*, which encodes a tyrosine kinase essential for cytokine and growth factor signaling, are found in a large proportion of patients with myeloproliferative neoplasms [9]. Ruxolitinib is a JAK1/JAK2 inhibitor approved for the treatment of myelofibrosis [10], and additional JAK2 inhibitors are in clinical development [11].

Detection of mutations in *EGFR*, *KRAS*, *BRAF*, *FLT3*, *NPM1*, and *JAK2* is most commonly accomplished by targeted tests that are designed to detect one or at most a small number of mutations in a single gene. However, NGS is gaining momentum as a complementary test for a number of reasons. Firstly, clinical trials for targeted cancer therapies rely on detection of mutations that are frequently not covered by existing targeted tests. In contrast to the laborious and lengthy process of validating and implementing a new molecular assay testing for one or a few mutations, NGS greatly simplifies the task of providing coverage of one of more additional mutations of interest. Secondly, targeted tests can provide misleading results. For example, the widely used FDA-approved cobas® *EGFR* Mutation Test only detects exon 19 deletion and L858R mutations, which together only comprise the mutations found in 85% of *EGFR*-mutated lung cancers [12]. In a significant proportion of cases, this test fails to identify therapeutically targetable mutations. Thirdly, targeted tests may fail to detect the very mutation they are designed to detect. Our group has recently reported a striking failure of two separate single-gene tests for the *BRAF* gene to detect a V600E mutation in a melanoma specimen [13]. The mutation was clearly demonstrated by concurrent NGS analysis. Finally, as has recently become apparent, tumors frequently harbor mutations that are therapeutically targetable but are not typically seen in that tumor type. Due to its massively parallel nature, NGS is very well-suited for detecting mutations in unexpected genes. One study reported a three-fold increased yield of clinically actionable mutations with NGS as compared to traditional molecular approaches targeting mutation hotspots [14].

Multiple recent studies have investigated the potential utility of NGS for detection of clinically actionable cancer mutations with encouraging results [14–25]. With the exception of one study [21], all demonstrated excellent performance of NGS on various platforms as measured by detection of point mutations and small insertions and deletions (indels). In many cases, additional potentially important variants were uncovered by NGS. All of the aforementioned studies were designed to validate clinical NGS pipelines that were not yet in clinical practice. As a consequence, they enrolled selected samples from previously examined specimens. With the exception of one commercially-sponsored study [14], in all cases a very limited number of samples was re-examined (range 13–61), often from only a single tissue type. While these important contributions confirm the potential usefulness of clinical NGS, they do not address the important question whether a well-validated NGS pipeline performs at an acceptable level in day-to-day clinical practice. Here we present a summative analysis of mutation results and quality control metrics obtained during the first year (March 2013 through March 2014) of clinical solid and liquid malignancy NGS carried out at the Center for Personalized Diagnostics at the University of Pennsylvania Health System. More than 900 specimens were submitted and processed during this time frame. We report that using our validated molecular and bioinformatics pipeline [26] with pre-determined tumor percentage and DNA quality cutoffs, we achieved excellent NGS data quality as determined by virtually perfect concordance between NGS and targeted single-gene tests for various genes in a large number of solid and liquid malignancy specimens.

## Materials and Methods

### Specimen Characteristics and Processing

Over the course of the study duration, 938 liquid and solid tumor specimens were submitted to the Center for Personalized Diagnostics (Table 1). Specimens were eligible for NGS if they passed the tumor percentage, DNA quality, and DNA quantity thresholds that had been determined at the time of the validation of the NGS assay, which preceded the study period. Briefly, specimens with <10% tumor were not eligible for NGS, because sequencing of samples with lower tumor percentages frequently yielded changes that were represented in fewer than five unique reads, making it difficult to distinguish true variants from sequencing artifacts. For similar reasons, DNA quality and quantity were judged to be insufficient, and the specimen was ineligible for NGS, if the DNA concentration was <1 ng/μL; the DNA concentration was <5 ng/μL with >20% DNA degraded; the DNA concentration was <50 ng/μL with >45% DNA degraded; or DNA degradation was >60%. Degraded DNA was defined as the proportion of DNA under 1000 bp in length.

**Table 1:** Characteristics of Specimens by Tumor Site.

Tumor site	Number of specimens submitted for NGS	Specimens with DNA quality or quantity inadequate for NGS analysis	Number of “shared” specimens (i.e., results are available from both NGS and targeted tests)
Lung	196	13 (6.6%)	101 (51.5%)
Brain	174	9 (5.2%)	6 (3.4%)
Bone Marrow	161	0	95 (59.0%)
Lymph Node	70	8 (11.4%)	32 (45.7%)
Peripheral blood	56	0	29 (51.8%)
Liver	36	4 (11.1%)	5 (13.9%)
Skin	23	2 (8.7%)	5 (21.7%)
Other	222	24 (10.8%)	31 (14.0%)

Tumor site is not necessarily tissue of origin. Please note that in contrast to solid tumor specimens, all liquid specimens (bone marrow and peripheral blood) were adequate for NGS processing.

To determine the tumor percentage and volume of solid tumors, hematoxylin- and eosin-stained tissue specimens were evaluated by an anatomic pathologist, and the region with the highest tumor burden was marked. Genomic DNA was extracted from fresh bone marrow or peripheral blood using the Gentra Puregene Cell Kit (Qiagen, Netherlands). For formalin-fixed, paraffin-embedded (FFPE) specimens, tissues were macro-dissected from 5 μM or 10μM slides. Scrapings were dewaxed with Qiagen Deparaffinization Solution and purified with Gentra Puregene Tissue reagents following the manufacturer’s protocol (Qiagen, Netherlands). DNA quantification was performed using the Qubit Broad Range assay following manufacturer’s protocols (Life Technologies, CA). Agilent Genomic TapeScreens were used following manufacturer’s protocols to assess the degree of DNA degradation (Agilent, CA).

### Targeted Molecular Testing

#### *EGFR*, *KRAS*, and *BRAF* Assays

The mutational status of *EGFR* exons 19 and 21 was determined using a laboratory-developed test (LDT) as previously described [27]. Briefly, genomic DNA was extracted from FFPE tissue and amplified with primers covering two regions, one that is commonly deleted in exon 19, and a part of exon 21 that encompasses codon 858. The L858R missense mutation in exon 21 creates a new *Sau*96I cleavage site within exon 21. The amplification products were digested with *Sau*96I and then separated by capillary electrophoresis.

*KRAS* mutations in codons 12 and 13 were assayed using a LDT as previously described [28]. Briefly, genomic DNA was extracted and amplified using primers designed to detect point mutations, hybridized to target-specific capture probes, and subjected to a bead assay (Lumina, TX).

*BRAF* mutations were assayed by pyrosequencing of an amplified portion of the *BRAF* gene including codon 600, as previously described [29].

#### *FLT3* Assay

DNA was extracted using the QIAamp DNA Blood Mini Kit (Qiagen, Netherlands). Mutation analysis of the *FLT3* gene was performed using multiplex PCR amplification with two sets of fluorescently labeled primers. For internal tandem duplication (ITD) detection, PCR was performed with the following primers: 5’-GCA ATT TAG GTA TGA AAG CCA GC-3’ (forward) and 5’-CTT TCA GCA TTT TGA CGG CAA CC-3’ (reverse); forward primers were labeled with 6-carboxyfluorescin (6-FAM), and reverse primers were labeled with VIC. An internal tandem duplication (ITD) was determined to be present if a product larger than the wild-type (329 bp) product was detected by capillary electrophoresis. Detection of the D835 mutation of *FLT3* (NM_004119.2: c.2503_2505) was based on the fact that this mutation abolishes an *Eco*RV cleavage site. PCR was performed with the following primers: 5’-GTA AAA CGA CGG CCA GCC GCC AGG AAC GTG CTT-3’ (forward) and 5’-CAG GAA ACA GCT ATG ACG ATA TCA GCC TCA CAT TGC CCC-3’ (reverse); forward primers were labeled with NED at the 5’ end. After *Eco*RV digestion, PCR products were analyzed by capillary electrophoresis using a 3500xL Genetic Analyzer (Life Technologies, NY). A D835 point mutation was indicated by the presence of a 129 bp fragment.

#### *NPM1* Assay

Total RNA was extracted using the QIAamp RNA Blood Mini Kit (Qiagen, Netherlands), reverse transcribed, and amplified in a multiplex PCR reaction using primers designed to detect common mutations in *NPM1* (NM_002520.4) using the Signature *NPM1* Mutations Assay (Asuragen, TX). Labeled PCR products were hybridized to target-specific capture probes covalently bound to fluorescent microspheres in a liquid bead array followed by analysis with a Luminex 100 (Luminex, TX). Interpretation was based on the mean fluorescence intensity (MFI) obtained from a minimum of 50 microspheres.

#### *JAK2* Assay

Genomic DNA was isolated from leukocytes using the QIAamp DNA Blood Mini Kit (Qiagen, Netherlands) and amplified using real-time PCR with primers flanking *JAK2* codon 617. Allelic discrimination between the normal sequence and the *JAK2* V617F (NM_004972 c.1849G>T) mutation was subsequently accomplished by simultaneous differential hybridization of two sequence-specific probes, each labeled with a different fluorescent marker (MutaScreen Assay, Qiagen, Netherlands).

### Next-Generation Sequencing and Bioinformatic Analysis

**Fig 1:**
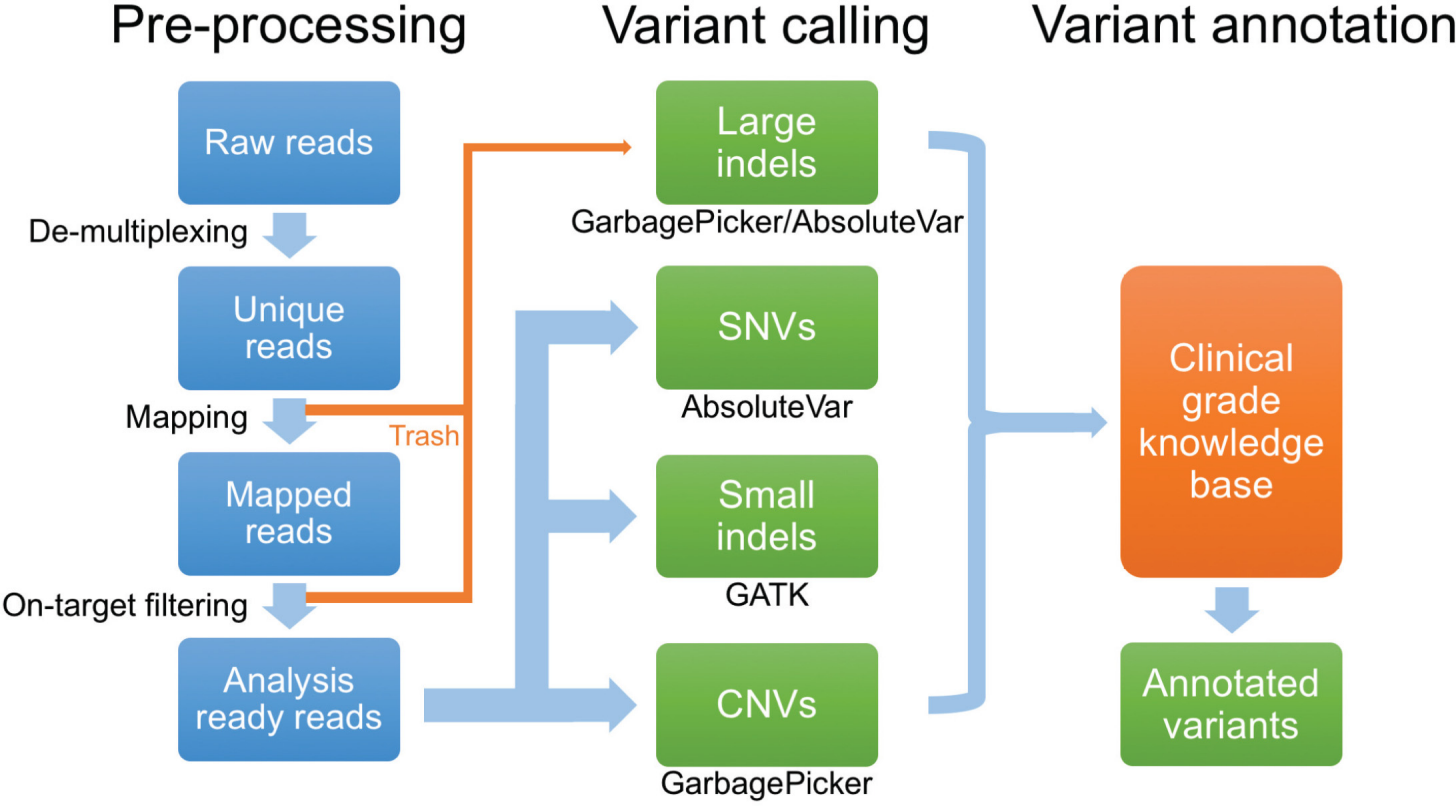
Next-generation sequencing data analysis pipeline. Data analysis occurs in three sequential stages, pre-processing of NGS reads, variant calling, and variant annotation. Of note, large indels are detected by an examination of reads that failed to map to target regions of the reference genome and are recovered from a pool of rejected reads (“Trash”). SNVs, single nucleotide variants. CNVs, copy number variation.

For solid tumors, target enrichment was performed with the TruSeq Amplicon Cancer Panel (Illumina, CA), a cancer gene panel consisting of 212 target amplicons covering mutation hotspots of 47 cancer genes. For hematologic malignancies, an in-house developed gene panel was utilized, which is composed of 382 amplicons covering 33 genes. For all successful sequencing runs, read depth was 250x at any given position, with 1000x mean coverage across the entire targeted sequence, and a Q30 at greater than 75% of reads. An in-house bioinformatics pipeline [26] was used to map reads, detect variants, and annotate them. Reads were de-multiplexed, mapped to the hg19 version of the human reference genome, filtered to remove off-target and poor-quality reads (Fig 1). Using custom scripts, four types of variants were extracted: single-nucleotide variants (SNVs), small indels, copy number variants, and large indels. Variants were then compared to an in-house developed knowledge base, which draws from publicly available sources such as PubMed, dbSNP database [30], COSMIC database [31], 1000 Genomes [32], and the Exome Variant Server (http://evs.gs.washington.edu). Using this knowledge base, variants were classified into 1 of 5 categories: disease associated mutation (DAM), likely pathogenic mutation (LPM), variants of uncertain significance (VUS), likely benign (LB), or benign (B). DAMs include mutations previously reported and associated with disease, including gain-of-function mutations in oncogenes (e.g., the canonical *KRAS* G12D mutation) and truncating mutations in known tumor suppressor genes. LPMs were classified as variants that had some evidence of disease association, such as case reports, but are not well described otherwise. Variants were classified as VUS if they had not been previously reported either as a disease-associated mutation or as a normal variant on the Exome Variant Server, but whose pathogenicity could not be established with certainty. Variants were classified as LB if there was no report of association with disease and if they occurred in regions of the gene not predicted to have pathologic consequences. Variants noted on the clinical report, which are the ones included in this analysis, included the DAM, LPM and VUS (not LB or B). All reported variants were manually reviewed using the Integrative Genomics Viewer (IGV) [33] by at least two pathologists.

### Ethics Statement

Patient data were analyzed anonymously in accordance with institutional practice guidelines. The institutional review board of the University of Pennsylvania determined this study to be exempt.

## Results

### Study Design and Specimen Characteristics

The University of Pennsylvania Health System began routine clinical NGS of solid tumors and hematologic malignancies in February of 2013. During validation of our NGS pipeline, we established thresholds for acceptable tumor percentage (10%) and DNA quantity and quality (see Methods for details).

**Fig 2:**
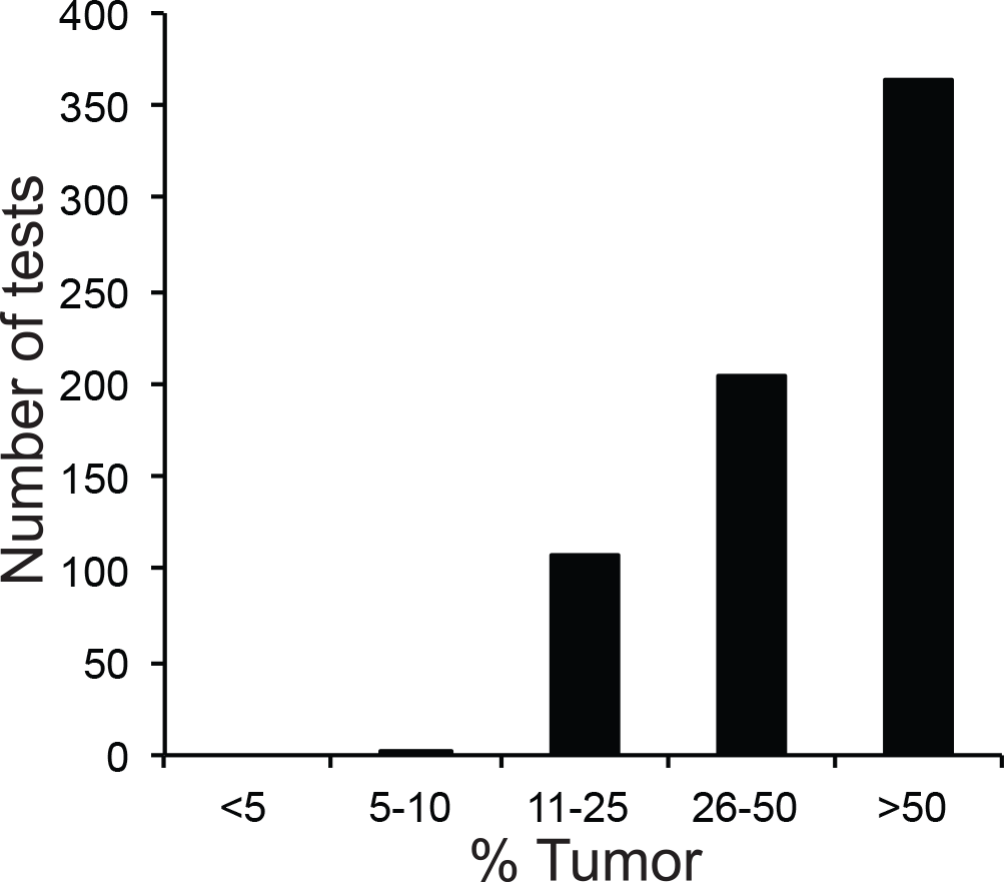
Tumor Percentage of Solid Tumor Specimens. Specimens were analyzed by next-generation sequencing (NGS) within the study period of March 1, 2013 and March 1, 2014.

For a large number of specimens, clinicians ordered targeted single-gene tests and NGS analysis on the same specimen (Table 1): this occurred frequently with pulmonary and hematological specimens, but relatively rarely with specimens derived from other sites such as brain. We reasoned that comparing results from targeted and NGS testing of all “shared” specimens (i.e. specimens that had been analyzed by both NGS and single-gene tests) would allow us to probe the robustness of our NGS pipeline. We therefore set out to compare NGS and targeted test results in specimens that underwent NGS analysis during the first year of operation of the NGS pipeline (March 1, 2013 through March 1, 2014). During this time, 938 specimens (717 solid and 221 hematologic) were submitted for NGS analysis. While the majority of solid tumor specimens were composed of >50% tumor cells (Fig 2), a fraction of solid tumors was not tested due to low tumor percentage. The most common tumor sites were lung, brain, bone marrow, lymph nodes, and peripheral blood (Table 1). A small fraction of solid tumor specimens could not be analyzed by NGS due to inadequate DNA quantity or quality (*n* = 60, 8.3%), however this was not an issue with peripheral blood and bone marrow specimens.

### Comparison of *EGFR* and *KRAS* Gene Mutations by NGS and Targeted Testing

**Fig 3:**
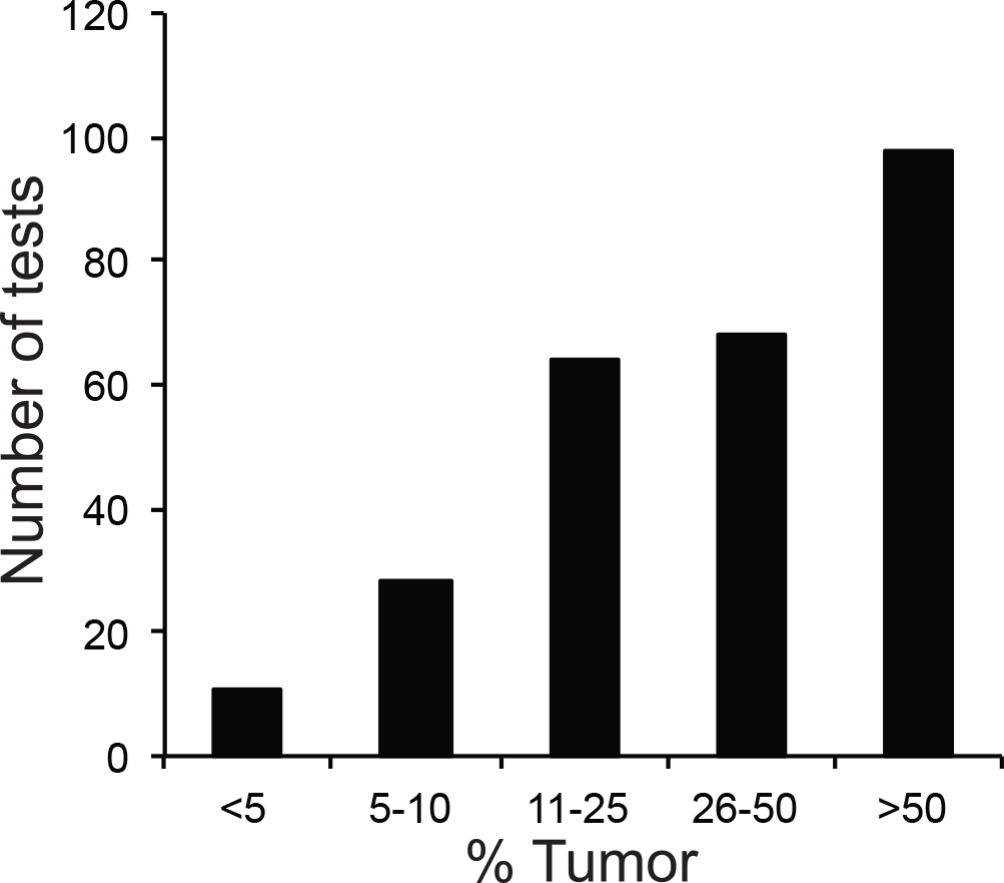
Tumor Percentage of Specimens Analyzed by Targeted *EGFR* Tests. Cases were placed into 5 bins (<5%, 5–10%, 11–25%, 26–50%, >50% tumor cells in specimen).

We were particularly interested in the performance of *EGFR* testing in our NGS assay, because mutations in this gene may be therapeutically targetable, and because it is often challenging to analyze, as DNA degradation and low tumor percentage are frequently encountered in lung cancer specimens. Since *KRAS* mutations were frequently evaluated alongside *EGFR* in cases of lung cancers at our institution, we also wanted to compare the performance of both methods on this gene. The tumor percentages for all specimens for which targeted *EGFR* mutation analysis was reported during the study period (*n* = 283) are shown in Fig 3. Approximately 10% of the cases evaluated by targeted testing had a tumor percentage below the cutoff for NGS.

**Fig 4:**
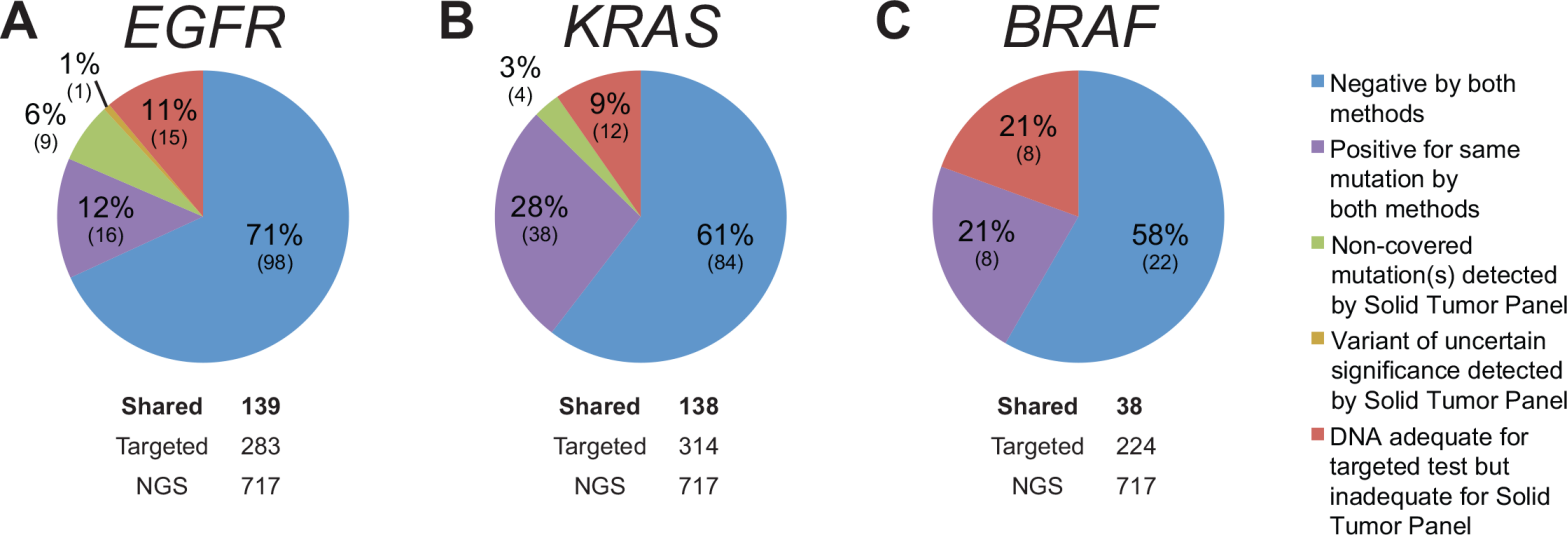
Concordance Analysis of Solid Tumor Specimens. The specimens shown were submitted for both NGS and targeted tests for *EGFR* (A), *KRAS* (B), and *BRAF* (C) mutations. Note that all mutations seen by targeted testing were also found by NGS when specimens with inadequate DNA quantity and/or quality are excluded.

We compared NGS and targeted testing in 139 specimens that had been tested for *EGFR* mutations (Fig 4A) and 138 that had been tested for *KRAS* mutations by both methods (Fig 4B). Among the shared specimens, all generated a result with the targeted single-gene methods. However, 15/139 (11%) of shared *EGFR*-tested and 13/138 (9%) of shared *KRAS*-tested specimens were excluded from NGS analysis due to insufficient DNA quality or quantity.

In the remaining specimens, all mutations detected by the targeted assays were also detected by NGS. Conversely, NGS identified a number of mutations that the targeted tests were not designed to detect. For example, our targeted *EGFR* mutation test only covers deletions in exon 19 and the L858R mutation in exon 21. Similarly, the targeted *KRAS* test only detects mutations in codons 12 and 13. In 10 *EGFR* shared cases (6%), additional pathogenic mutations were detected by NGS. In two of these cases, the T790M mutation was found, which predicts resistance to TKI therapy [4]. Additionally, NGS detected *EGFR* amplification in five cases; the predictive value of this copy number alteration in the context of TKI therapy is currently unclear. In four *KRAS* shared cases (3%), NGS detected mutations in codon 61, which predict resistance to TKI therapy [34].

### Comparison of *BRAF* Gene Mutations by NGS and Targeted Testing

The *BRAF* single-gene test was performed less frequently in parallel with solid tumor NGS than the *EGFR* or *KRAS* tests, because thyroid fine-needle aspiration samples, for which *BRAF* testing was frequently ordered, were not originally validated for the NGS assay. Of 224 specimens that were tested with targeted *BRAF* tests, 38 were also analyzed by NGS (Fig 4C). Among the shared specimens, all generated a result with the targeted *BRAF* test. In contrast, 8/38 shared cases (21%) could not be analyzed by NGS due to poor DNA quality or inadequate DNA quantity. All remaining shared specimens showed perfect concordance between NGS and targeted testing.

### Comparison *FLT3*, *NPM1*, and *JAK2* Mutations by NGS and Targeted Testing

**Fig 5:**
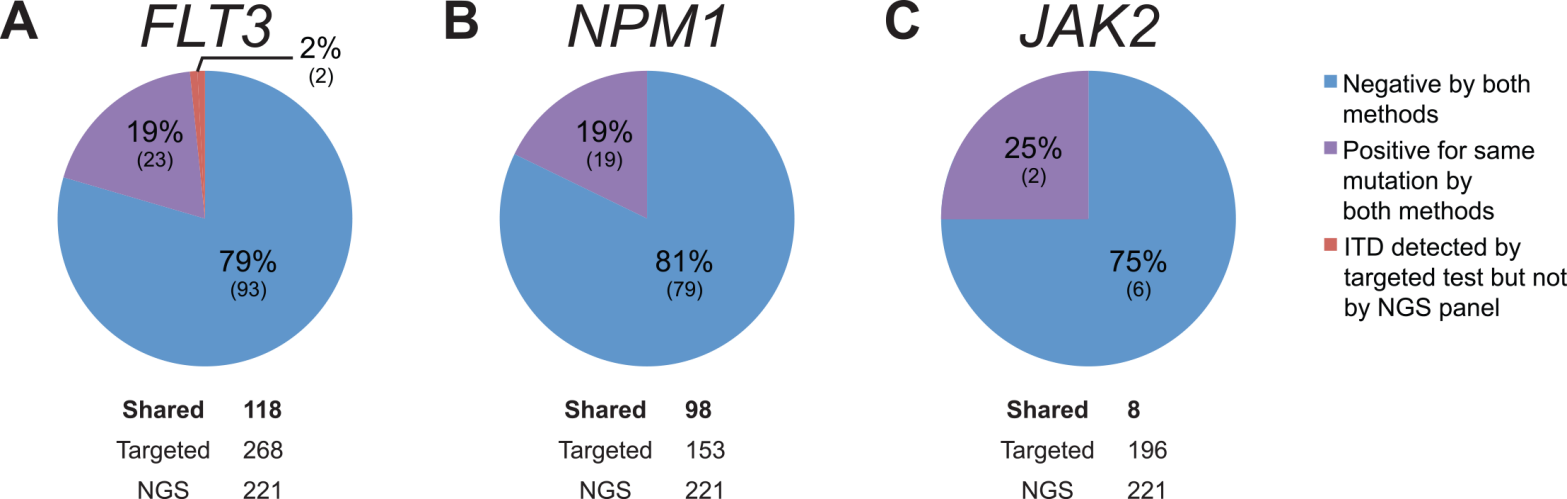
Concordance Analysis of Hematologic Malignancy Specimens. The specimens shown were submitted for both NGS and targeted tests for *FLT3* (A), *NPM1* (B), and *JAK2* (C) mutations. Note that all samples were adequate for testing by both single-gene assays and NGS.

**Fig 6:**
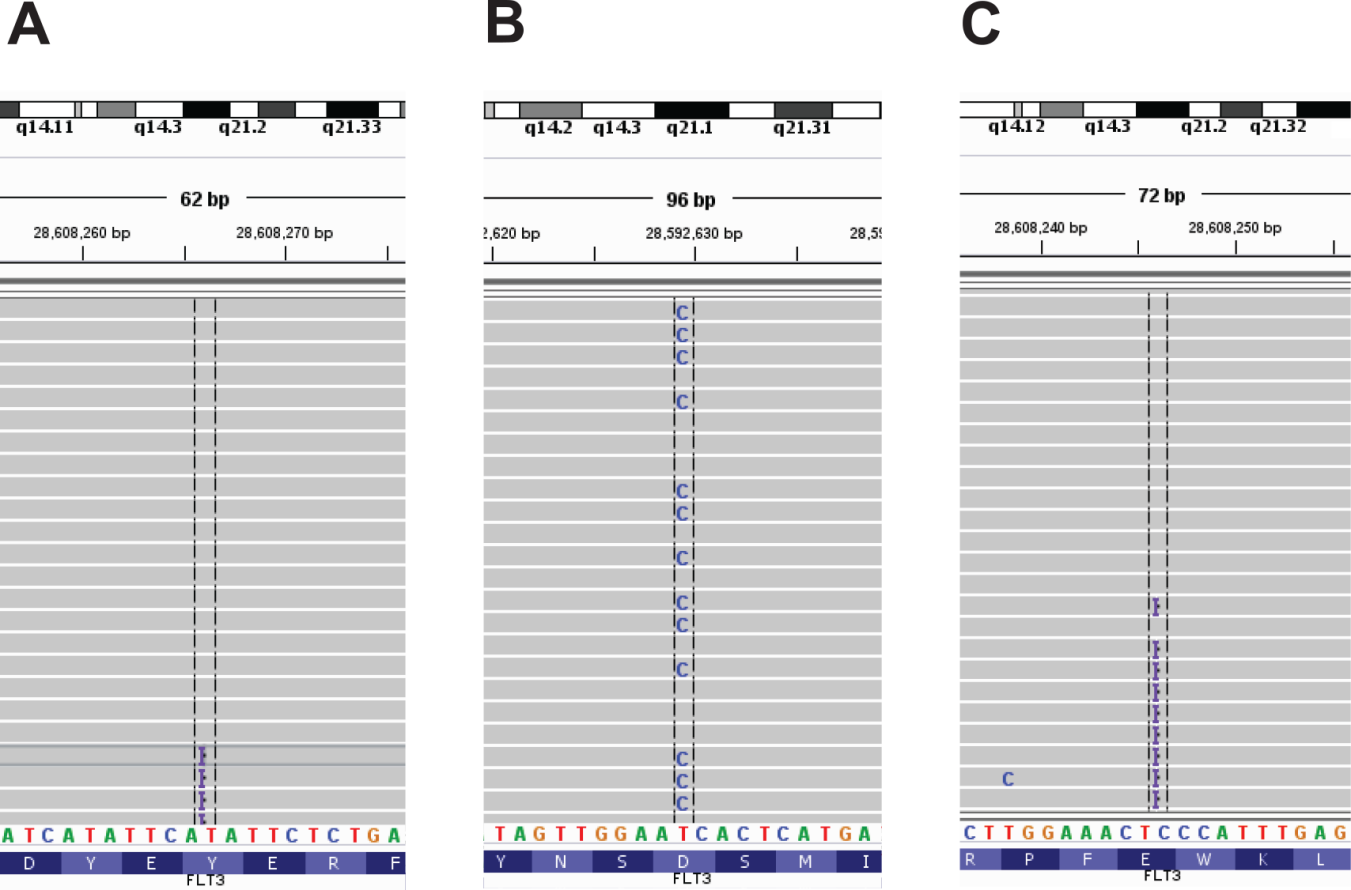
Two Specimens with Low-Allele Frequency *FLT3* Internal Tandem Duplications. In one specimen (A and B), a 24 bp internal tandem duplication (ITD) was seen in 7 out of 529 reads for an allele frequency of 1.3%. (A) Four of the reads containing insertions (purple bars) are shown using the Integrative Genomics Viewer. This specimen additionally harbored a *FLT3* D839G mutation in 45% of reads (B). A second specimen (C) harbored a 33 bp *FLT3* ITD in 12 out of 739 reads, for an allele frequency of 1.6%. Nine of the reads carrying an ITD are pictured.

Of 221 hematologic specimens tested by NGS during the study period, 118 were also tested with the *FLT3*, and 98 with the *NPM1* targeted tests. In two cases, the targeted *FLT3* test detected an internal tandem duplication (ITD) that was not automatically called by the NGS analysis pipeline (Fig 5A). However, manual review of the sequencing data demonstrated ITD mutations at allele frequencies of 1.3% and 1.6% (Fig 6A and 6C, respectively). In one of these two cases, the hematologic malignancy NGS panel additionally detected a pathogenic *FLT3* D839G (c. 2516A>G) mutation (Fig 6B); this mutation was not detected by the *FLT3* single-gene test that is designed to detect only ITDs and D835 mutations.

In the remainder of *FLT3* shared cases, and also in all *NPM1* shared cases (Fig 5B), NGS and targeted tests were concordant. Additionally, a small number of specimens (*n* = 8) that had been tested for *JAK2* mutations by both modalities were analyzed and showed complete concordance (Fig 5C).

## Discussion

In this study, we report the properties of solid and liquid malignancy specimens processed during the first year of clinical oncologic NGS performed within the University of Pennsylvania Health System. We found that when we adhered to two predetermined quality control metrics, i.e., tumor percentage and DNA quantity and quality, we achieved excellent NGS data as determined by virtually perfect concordance between NGS and targeted, single-gene testing. While a number of recent studies have confirmed the potential utility of clinical NGS for oncology [14–25], there have been none, to our knowledge, that have evaluated the quality of data generated during day-to-day practice at a clinical oncologic sequencing facility. Additionally, in contrast to previous validation studies, we examined a larger number of specimens from a greater variety of tissues, and our data is therefore less subject to sample selection bias and may also more accurately reflect the expected annual case volume and distribution of tissue types encountered at a major academic medical center.

Solid tumors and hematologic malignancy specimens differed considerably in their performance across the established quality control measures. We found that a sizable fraction of solid but not liquid specimens yielded DNA of insufficient quality or quantity for NGS testing. With respect to specimens tested for *EGFR* and *KRAS* (predominantly lung), this was likely largely due to formalin fixation, which degrades DNA through cross-linking as well as other less well understood mechanisms [35]. One potential approach to reduce the number of samples that are currently rejected from NGS analysis might be to determine the amplifiability of extracted genomic DNA, for example by using the human genomic DNA quantitation and quality control assay by Kapa Biosystems (Wilmington, MA).

Our study highlights the complementarity of NGS and targeted tests for mutation detection. In a number of instances, NGS detected clinically important mutations that were not captured by targeted assays. For example, the *EGFR* test specifically interrogates potential exon 19 deletions and L858R mutations, which constitute about 85% of EGFR mutations in lung cancers [12]. In 9 shared cases, *EGFR* mutations were found in different regions of the gene by the NGS assay (Fig 4A). Similarly, clinically important *KRAS* mutations occur in codons 12, 13, and 61, but only codons 12 and 13 were evaluated by the in-house *KRAS* single-gene test. In 4 shared cases, *KRAS* mutations in codon 61 were detected by NGS only.

Targeted assays generally outperformed NGS in specimens with low tumor percentage, DNA quantity, or quality. In fact, we identified actionable mutations by targeted analysis in multiple cases that were unable to be analyzed by NGS (data not shown). In two specimens, a *FLT3* ITD mutation, which was not automatically called by the NGS pipeline, was readily detected by the targeted assay. While *FLT3* ITDs can be challenging to detect by NGS due to the complex structure of the mutation [36], the problem in these two cases was that the allele frequency fell below the validated threshold for automatic detection of 4%. However, manual inspection of the *FLT3* exon 14 and the flanking intronic sequence using the Integrative Genomics Viewer (IGV) [33] clearly showed the presence of ITD mutations in both cases (Fig 6A and 6C). The detection of NGS for indel mutations, in contrast to single nucleotide variants, is not fundamentally limited by PCR artifacts and sequencing errors. Therefore, we validated our pipeline for the detection of *FLT3* ITDs with allele frequencies of 1%, and we altered the indel allele frequency calling threshold specifically for *FLT3* ITDs. Reanalysis of the two *FLT3* cases with updated parameters, which was part of the revalidation study, revealed the expected results. These findings highlight the importance of manual review of the sequencing data.

Low tumor percentage was also limiting in solid tumor cases. Approximately 10% of specimens submitted for *EGFR* mutation testing were found to contain less than 10% tumor (Fig 3), thereby failing tumor percentage requirements for NGS testing. All of these specimens generated a result with the targeted assay. It should be noted, however, that as NGS methodologies continue to mature, the ability of NGS to detect single nucleotide variants in specimens with lower tumor percentage will improve. Rare variant detection by NGS is hampered by the high rate of amplification and sequencing errors, which can approach 1% for single nucleotide variants [37]. Various approaches to increase analytic sensitivity for rare variants by NGS have recently been described, including barcoding of each DNA fragment before amplification [38–40], barcoding of both strands of each fragment [41], and generation of multiple linked tandem copies of each DNA fragment by rolling circle amplification [42]. In particular, the latter method has been shown to improve sensitivity of rare variant detection by more than 100-fold without introducing excessive computational inefficiency.

There are a number of limitations to this study. First, only genes for which both NGS analysis and single-gene tests are performed at our institution were included in the analysis. While the genes examined in this study currently represent the essential core of cancer gene testing, it is possible that NGS might function less reliably with certain other genes or specific mutations that were not assessed. Of note, our NGS pipeline produced excellent results for the challenging genes *EGFR* and *FLT3*. We therefore expect that most other genes covered by our cancer panels generate NGS data of similarly high quality. Second, not all specimens that were submitted for NGS analysis were also tested by single-gene methods. For example, only 3.4% of brain tumor specimens submitted for NGS were at the same time examined by targeted tests, and therefore our conclusions may not necessarily extend to all tumors at this time. Additionally, the specimens tested did not contain the entire spectrum of mutations that can be evaluated by our targeted tests. Finally, our findings may not be generalizable to other platforms, especially those that utilize more complex gene panels.

Recently issued recommendations for validation and quality control of clinical NGS data [43] include monitoring of quality metrics (e.g., sequencing quality scores, depth of coverage, uniformity of coverage, mapping quality), proficiency testing, and confirmation of actionable results by independent methods. Multiple studies that appeared after these recommendations were published have established that particularly for solid tumors, tumor percentage and DNA quality are important additional metrics [14,22,23], and we found this as well in our validation studies (not shown). Proficiency testing for cancer NGS is not yet available but is currently being developed by the College of American Pathologists (CAP).

A proposed requirement to confirm actionable mutations by independent molecular methods [43] has been called into question [23]. Since single-gene tests such as Sanger sequencing may not have inherently greater sensitivity than a well-scaled NGS pipeline [15,44], verification by these methods might not improve the accuracy of test results. Accordingly, very recent guidelines by the College of American Pathologists leave the decision when and how to perform confirmatory testing of NGS results to the clinical laboratory [44]. Our *EGFR* single-gene test had greater analytical sensitivity than NGS, detecting mutations in samples with well under 10% tumor. However, our finding that within pre-defined tumor percentage and DNA quality cutoffs NGS showed perfect concordance confirms the notion that it is unnecessary to confirm each actionable mutation detected via NGS by a single-gene test. In addition, single-gene tests frequently do not cover important disease-associated mutations and in some instances fail to detect the very mutations they target [13].

**Fig 7:**
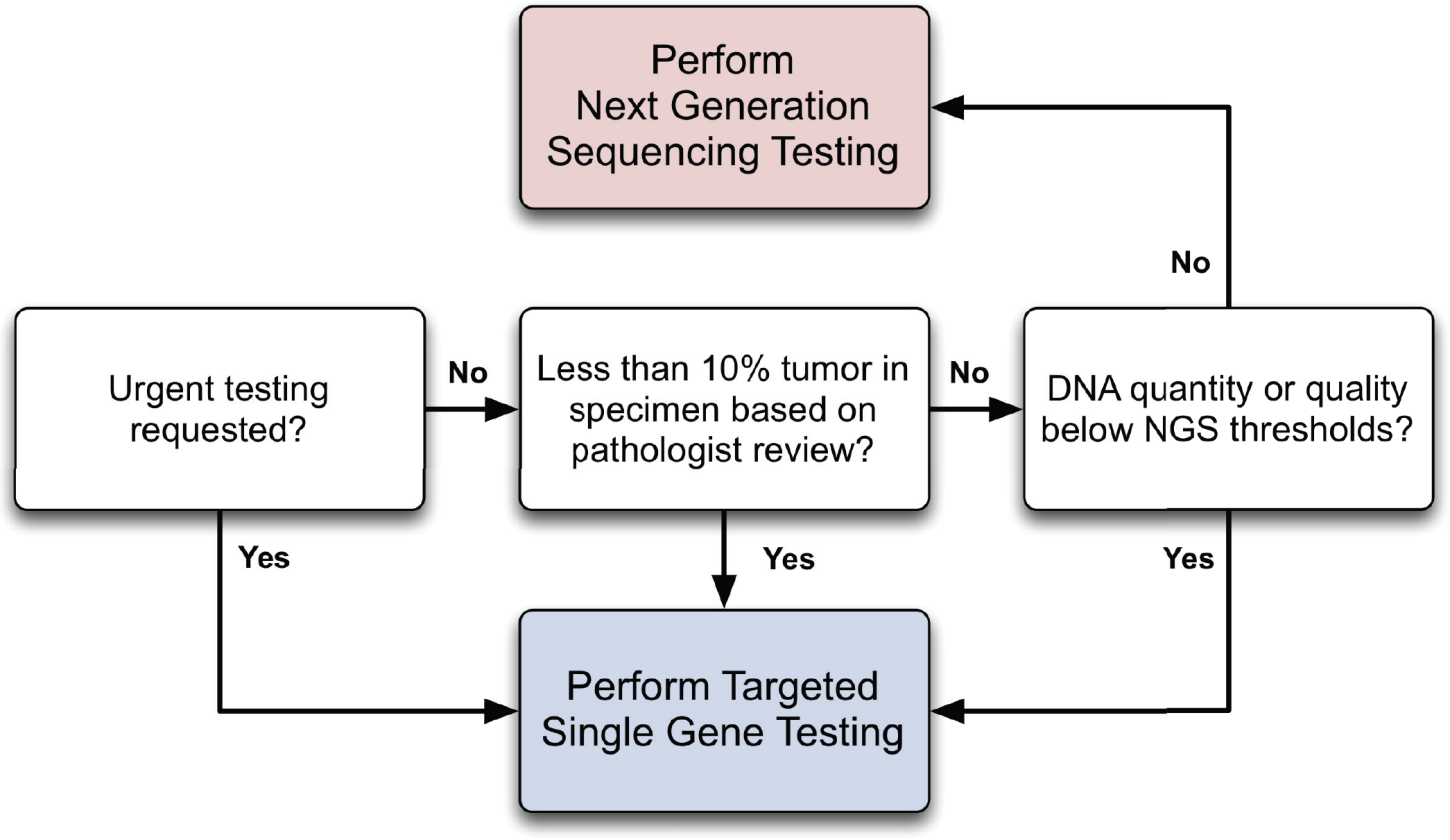
Proposed Workflow for NGS and Single-Gene Assays. Three main decision points are highlighted. Specimens requiring an urgent turnaround time are routed directly for single-gene testing (possibly followed by NGS). Additionally, single-gene testing is performed on samples with less than 10% tumor or DNA inadequate for NGS (i.e., degraded or low quantity). In samples not meeting any of the above criteria, NGS is performed instead of single-gene testing. NGS results do not require confirmation by single-gene testing.

Based on these considerations, we propose a workflow that integrates NGS as an adjunct diagnostic modality for solid and liquid neoplasms (Fig 7). The typical turn-around time of the NGS assay at the time of the study was around 7–10 days from receipt in the sequencing facility to the time the final report was signed out in the electronic medical record. Turnaround is generally faster for targeted tests, and thus it is advantageous to perform targeted tests, perhaps in addition to NGS, in clinically urgent situations. Specimens with low tumor percentage or DNA quantity or quality should be subjected to targeted tests that we found to be more analytically sensitive than NGS. On the other hand, when DNA quality and quantity is adequate and a turnaround time of 7–10 days is acceptable, NGS holds clear advantages, including increased clinical sensitivity within targeted genes of interest and additional information from other covered genes on the panel. In these cases, our data suggest that targeted tests may be safely omitted.

## Supporting Information

S1 File Raw Data for Comparison Analysis. (XLSX)
